# Association between early sexual initiation and sexually transmitted infections among Peruvian reproductive-age women

**DOI:** 10.3389/fpubh.2023.1191722

**Published:** 2023-09-18

**Authors:** Jhosuny Perez-Fernandez, Diego O. Arroyo-Velasco, Mariella R. Huaman, Sarai G. Chavez-Bustamante, Anita P. Llamo-Vilcherrez, Carolina J. Delgado-Flores, Carlos J. Toro-Huamanchumo

**Affiliations:** ^1^School of Medicine, Universidad de San Martín de Porres, Chiclayo, Peru; ^2^Sociedad Científica San Fernando, Universidad Nacional Mayor de San Marcos, Lima, Peru; ^3^Sociedad Científica Médico Estudiantil Continental, Universidad Continental, Huancayo, Peru; ^4^Grupo Peruano de Investigación Epidemiológica, Unidad de Investigación para la Generación y Síntesis de Evidencias en Salud, Universidad San Ignacio de Loyola, Lima, Peru; ^5^Carrera de Farmacia y Bioquímica, Facultad de Ciencias de la Salud, Universidad Científica del Sur, Lima, Peru; ^6^Unidad de Investigación para la Generación y Síntesis de Evidencias en Salud, Universidad San Ignacio de Loyola, Lima, Peru; ^7^OBEMET Centro de Obesidad y Salud Metabólica, Lima, Peru

**Keywords:** sexual behavior, health risk behaviors, sexually transmitted diseases, women’s health, Peru

## Abstract

**Background:**

Sexually transmitted infections (STIs) are a serious public health problem worldwide, especially among reproductive-age women. The early sexual onset of sexual intercourse (EOSI) has been suggested as a risk factor, although there is no data at the national level.

**Objective:**

To evaluate the association between EOSI and STIs in Peruvian women of childbearing age.

**Methods:**

Analytical cross-sectional study with secondary data analyzes of the Peruvian Demographic and Family Health Survey 2018. The outcome was the presence of STIs in the last 12 months and the exposure variable was EOSI (age < 15 years at the time of their first sexual experience). To evaluate the association of interest, crude and adjusted prevalence ratios (aPRs) were calculated using generalized linear models with Poisson family and logarithmic link function.

**Results:**

We analyzed data from 31,028 women of childbearing age. The 11.3% reported having STIs in the last 12 months and 20.2% of the participants had an EOSI. After adjusting for potential confounders, we found that EOSI was associated with STIs (aPR: 1.27; 95% CI: 1.08–1.50; *p* = 0.005). When conducting stratified analysis by area of residence and number of sexual partners, this association was maintained in women living in urban areas (aPR: 1.36; 95% CI: 1.11–1.66; *p* = 0.003) those who did not report having a history of multiple sexual partners (aPR: 1.27; 95% CI: 1.08–1.51; *p* = 0.005), and those in the middle (aPR: 1.42; 95% CI: 1.03–1.97; *p* = 0.034) and highest (aPR: 2.12; 95% CI: 1.33–3.39; *p* = 0.002) wealth quintiles.

**Conclusion:**

Among reproductive-age women from Peru, EOSI was associated with STIs, especially in women living in urban areas, with no history of multiple sexual partners, and belonging to the middle to higher wealth index. The implementation of measures to prevent EOSI and fostering appropriate sexual health counseling for women with EOSI is advised.

## 1. Introduction

Sexually transmitted infections (STIs) represent a significant public health threat globally, primarily affecting family units, societal health, and individuals between the ages of 10–25 years (1–3). According to the World Health Organization (WHO), one million people contract an STI every day, predominantly chlamydia (127 million), gonorrhea (87 million), syphilis (6.3 million), trichomoniasis (156 million), and infections caused by hepatitis B virus, herpes simplex virus, human immunodeficiency virus (HIV), and human papillomavirus (HPV) (4). Notably, a strong association has also been observed between STIs and cancer (5–7), which also escalates healthcare costs (8, 9).

Evidence indicates that individuals exhibiting high-risk behaviors, such as frequent alcohol and tobacco use (10–12) drug use (13), engagement in unprotected sexual practices, or having multiple sexual partners (3, 14, 15) are at an increased risk of contracting STIs. Other contributing factors include exposure to sexual and physical violence (16), low socioeconomic status, low education level, rural place of origin, single marital status (12–15, 17), and early initiation of sexual activity (14, 18).

Early onset of sexual intercourse (EOSI) is defined as having a first sexual experience before the age of 15 years (18). Factors associated with EOSI reported in literature encompass substance use disorders (19–21), deficient family planning knowledge (21), inadequate family support (22), and a history of childhood abuse (23). This tends to amplify the occurrence of unwanted pregnancies, abortions, financial burdens, and STIs (10, 14, 16). In fact, studies conducted in Asia (14), Africa (24, 25), and America (26–30) have reported that young people with an EOSI are more likely to exhibit symptoms and receive an STI diagnosis during their lifetime. In Peru, (31) a study conducted in the province of Alto Huallaga (Huanuco, Peru) by Gomez et al. (27) found no statistically significant association between the age of first sexual intercourse (≤13 years) and STIs in their multivariable model. It should be noted that this study was restricted to a primary healthcare center’s population and solely included adolescents and young adults aged 18–24.

Globally, the average age of the onset of sexual activity is unknown, as most reports of sexual activity are more focused on selective groups (32–34). In Peru, it has been reported that half of the women between 25 and 49 years experienced their first sexual intercourse at 18.5 years (35). Furthermore, approximately 13% of women reported an STI or symptoms such as vaginal discharge or genital ulcers in 2017 (36). Therefore, despite the relevance and the potential connection between EOSI and STIs, there remains a lack of research in this area. Hence, this study aimed to evaluate the association between EOSI and STIs among reproductive-age women in Peru.

## 2. Materials and methods

### 2.1. Study design and data collection

We conducted an analytical cross-sectional study with secondary data analyzes. The study data were obtained from the Demographic and Family Health Survey (ENDES, Spanish acronym), which was carried out in 2018 (February to December) (35). ENDES, conducted annually by the National Institute of Statistics and Informatics (INEI, Spanish acronym), is a sample survey stratified by clusters representative at the national level that provides information on the health indicators of the Peruvian population. It comprises three questionnaires (Household, Individual women, and Health), and the data are collected in person by certified personnel. For this study, we used the Individual Questionnaire for Women, particularly the sections Medical Records; Reproduction; Birth History; Marriage; Knowledge and Attitude Regarding AIDS and Other STIs; HIV/AIDS; and Contraceptive Use. Additional information on the methodology of the survey can be found on the ENDES website.^1^

### 2.2. Sample population and context

The original ENDES 2018 data set included 39,745 women of childbearing age (15–49 years). This study excluded the data of 3,730 participants who had not yet commenced their sexual activityt, 174 participants who had their first sexual intercourse with their first partner and did not indicate their age at the time of the occurrence, and 4,813 participants with missing data for the variables of interest of this study. Finally, the analysis sample comprised 31,028 women aged 15–49 years who had already initiated their sexual activity. It is important to mention that our study only includes women, as complete information is only available for this population (i.e., there is no “Individual Men’s Questionnaire”).

### 2.3. Dependent variable

The main outcome of this study was the presence of STIs in the last 12 months. For its development, the RE758081 file of the ENDES 2018 microdata was considered, which included information on the knowledge, attitudes, opinions, and behaviors of women surveyed regarding STIs and AIDS. Variables with the codes V763A, V763B, and V763C were used to determine if the interviewee had any STI. The questiorans for the same were “In the last 12 months, have you been diagnosed with any STIs?0”; “During the last 12 months, have you had any sore or ulcer on your genitals?”; and “During the last 12 months, have you had any foul-smelling genital discharge or secretion?”. A new variable was created from these questions and dichotomized into Yes (when the participant answered yes to any of the questions) and No/Do Not Know.

### 2.4. Exposure

The primary independent variable whose association with the main outcome was evaluated in this study was the EOSI, which was constructed using data from the interviewee’s self-report of their age (in years) at the time of their first sexual experience. The cutoff age for the categorization of the study participants was set as below and equal to or above 15 years (22, 37–39).

### 2.5. Other variables

Other independent variables were also considered in the study, including age categories (15–19, 20–24, 25–29, 30–39; 40–49 years), marital status (unmarried; married/cohabiting), level of education (preschool/primary; secondary; professional/undergraduate/postgraduate), currently employed (yes; no), wealth index (very poor; poor; average; rich; very rich), region of origin (metropolitan Lima; rest of the coast; mountains; rainforest), area of residence (rural; urban), parity (childless; 1–2 children; >3 children), multiple sexual partners (yes [≥2 partners]; no [<2 partners]), and use of contraception (no contraception; condom; other).

### 2.6. Statistical analysis

The ENDES data were downloaded and filtered using the study variables as a reference. The statistical analysis of the data was performed by the Stata v16.0 statistical package (Stata Corporation, College Station, Texas, United States). The complex sample structure of the data was adjusted using the *svy* command, considering the sampling units, strata, and corresponding weighting factors for women of childbearing age. For univariate analysis, the categorical variables were summarized by absolute frequencies and weighted proportions, with a corresponding 95% confidence intervals (CI). The Chi-square test of independence along with Rao–Scott correction was used for comparing the independent variables and STI in the last 12 months.

To evaluate the association of interest, crude prevalence ratios and adjusted prevalence ratios (aPRs) were calculated using generalized linear models (GLMs) with Poisson family and logarithmic link function. The adjusted model followed an epidemiological approach and was adjusted for all the potentially confounding variables (age, marital status, level of education, wealth index, region of origin, residence, and use of contraception). All the estimates were reported with their corresponding 95% CIs, and *p* < 0.05 was considered significant.

Finally, interactions with the area of residence, multiple sexual partners, and wealth index were assessed using the adjusted Wald test (*testparm* command). Since the results were significant for these interactions, the final models were presented in a stratified manner.

### 2.7. Ethical aspects

The research protocol was reviewed and approved by the Institutional Review Board of the Universidad de San Martín de Porres (Record No. 991-2020-CIEI-FMH-USMP). This was an analytical study of the secondary database, which can be freely availed and accessed on the INEI website. This database is anonymous and does not reveal the identification of the participants.

This study offers both scientific and social benefits by addressing a gap in research regarding the association between early onset of sexual intercourse (EOSI) and sexually transmitted infections (STIs) among reproductive-age women in Peru. The findings can refine intervention strategies, enhance health policies, and guide future research to mitigate STI prevalence. Furthermore, the study aids in increasing awareness about STI risk factors, including EOSI, facilitating the development of targeted educational programs and community outreach efforts that promote safer sexual practices and ultimately improve public health and societal well-being.

## 3. Results

### 3.1. Sociodemographic characteristics of the study population

The study sample included 31,028 women of childbearing age, with majority of the participants in the 30–39 years age group (33.5%), followed by the 40–49 years age group (29.8%). In addition, 20.2% of the participants had an EOSI and 11.3% reported having STIs. The other sociodemographic variables are presented in Table 1.

**Table 1:** Sociodemographic characteristics of the study population (*n* = 31,028).

Variables	*n* (weighted %)
Age (years)	
15–19	1922 (5.8)
20–24	5,199 (14.2)
25–29	5,964 (16.7)
30–39	11,158 (33.5)
40–49	6,785 (29.8)
Area of residence	
Urban	22,059 (82.0)
Rural	8,969 (18.0)
Wealth index	
Very poor	8,227 (16.8)
Poor	7,787 (20.8)
Average	6,386 (21.3)
Rich	4,941 (20.8)
Very rich	3,687 (20.3)
Marital status	
Unmarried	8,048 (35.0)
Married/cohabiting	22,980 (65.0)
Region of origin	
Metropolitan Lima	4,217 (37.6)
Rest of the coast	9,447 (25.3)
Mountains	10,037 (23.8)
Rainforest	7,327 (13.3)
Level of education	
Preschool/Primary	7,079 (19.3)
Secondary	13,329 (39.7)
Professional/Undergraduate/Postgraduate	10,620 (41.0)
Currently employed	
No	11,621 (32.2)
Yes	19,407 (67.8)
Use of contraception	
No methods used	9,733 (38.0)
Condom	3,626 (13.3)
Others	17,669 (48.7)
Parity	
No children	3,408 (21.2)
1–2 children	16,526 (48.7)
3≥ children	11,094 (30.1)
MSP	
No	30,688 (98.7)
Yes	340 (1.3)
EOSI	
No	23,531 (79.8)
Yes	7,497 (20.2)
STI	
No	27,762 (88.7)
Yes	3,266 (11.3)

MSP, Multiple Sexual Partners; EOSI, =Early Onset of Sexual Intercourse; STI, Sexually Transmitted Infection.

### 3.2. Characteristics of the study population according to their history of STIs

We found that the prevalence of STIs was significantly higher among women aged 15–19 years (*p* = 0.004), those living in urban areas (*p* = 0.035), those from metropolitan Lima (*p* < 0.001), those with secondary education (*p* = 0.007), those who had previously used condoms as a contraceptive method (*p* = 0.017), and those with a history of multiple sexual partners (*p* < 0.001). Regarding EOSI, 13.5% of the women in this category reported having an STI in recent months (*p* = 0.003) (Table 2).

**Table 2:** Bivariate analysis of STIs and the variables (*n* = 31,028).

Variables	STI		*p* ^a^
	Yes (3266)	No (27762)	
	*n* (weighted %)	*n* (weighted %)	
Age (years)			0.004
15–19	225 (15.5)	1,697 (84.5)	
20–24	581 (12.6)	4,618 (87.4)	
25–29	661 (11.7)	5,303 (88.3)	
30–39	1,162 (11.3)	9,996 (88.7)	
40–49	637 (9.6)	6,148 (90.4)	
Area of residence			0.035
Urban	2,363 (11.5)	19,696 (88,5)	
Rural	903 (10.1)	8,066 (89.9)	
Wealth index			0.585
Very poor	819 (10.2)	7,408 (89.8)	
Poor	846 (11.3)	6,941 (88.7)	
Average	708 (11.8)	5,678 (88.2)	
Rich	562 (11.9)	4,379 (88.1)	
Very rich	331 (11.0)	3,356 (89.0)	
Marital status			0.891
Unmarried	873 (11.2)	7,175 (88.8)	
Married/cohabiting	2,393 (11.3)	20,587 (88.7)	
Region of origin			<0.001
Metropolitan Lima	553 (13.1)	3,664 (86.9)	
Rest of the coast	872 (9.0)	8,575 (91.0)	
Mountains	1,221 (12.0)	8,816 (88.0)	
Rainforest	620 (9.3)	6,707 (90.7)	
Level of education			0.007
Preschool/primary	668 (9.8)	6,411 (90.2)	
Secondary	1,537 (12.6)	11,792 (87.4)	
Professional/Undergraduate/Postgraduate	1,061 (10.7)	9,559 (89.3)	
Currently employed			0.115
No	1,131 (10.5)	10,490 (89.5)	
Yes	2,135 (11.7)	17,272 (88.3)	
Use of contraception			0.017
No methods used	1,012 (10.4)	8,721 (89.6)	
Condom	446 (13.9)	3,180 (86.1)	
Others	1808 (11.3)	15,861 (88.7)	
Parity			0.217
No children	426 (12.6)	2,982 (87.4)	
1–2 children	1723 (10.9)	14,803 (89.1)	
3 ≥ children	1,117 (11.0)	9,977 (89.0)	
MSP			< 0.001
Yes	81 (27.5)	259 (72.5)	
No	3,185 (11.1)	27,503 (88.9)	
EOSI			0.003
Yes	849 (13.5)	6,648 (86.5)	
No	2,417 (10.7)	21,114 (89.3)	

a Chi-square test.

MSP, Multiple Sexual Partners; EOSI, Early Onset of Sexual Intercourse; STI, Sexually Transmitted Infection.

### 3.3. Association between the EOSI and STIs

Table 3 shows the crude and adjusted model for the association of interest. In all the sample, after adjustinf for potential confounders, the EOSI was found to be positively associated with STIs (aPR: 1.27; 95% CI: 1.08–1.50; *p* = 0.005). When conducting stratified analysis by area of residence, number of sexual partners and wealth index, this association was maintained in women living in urban areas (aPR: 1.36; 95% CI: 1.11–1.66; *p* = 0.003), those who did not report having a history of multiple sexual partners (aPR: 1.27; 95% CI: 1.08–1.51; p = 0.005), and those in the middle (aPR: 1.42; 95% CI: 1.03–1.97; *p* = 0.034) and highest (aPR: 2.12; 95% CI: 1.33–3.39; *p* = 0.002) wealth quintiles. The association was marginally statistically significant in the subgroup within the second highest wealth quintile (aPR: 1.49; 95% CI: 0.93–2.38; *p* = 0.095).

**Table 3:** Generalized linear models for the association between EOSI and STIs.

Exposure: EOSI	Crude model			Adjusted model		
	cPR	CI 95%	*p*	aPR	CI 95%	*p*
Analysis for all the sample						
No EOSI	Ref.			Ref.		
Yes EOSI	1.26	1.09–1.47	0.002	1.27^a^	1.08–1.51	0.005
Analysis for the subgroup living in urban areas						
No EOSI	Ref.			Ref.		
Yes EOSI	1.41	1.18–1.69	<0.001	1.36^b^	1.11–1.66	0.003
Analysis for the subgroup living in rural areas						
No EOSI	Ref.			Ref.		
Yes EOSI	0.89	0.74–1.07	0.223	1.00^b^	0.83–1.21	0.996
Analysis for the subgroup with history of MSP						
No EOSI	Ref.			Ref.		
Yes EOSI	0.92	0.43–1.99	0.833	0.75^a^	0.35–1.63	0.475
Analysis for the subgroup with no history of MSP						
No EOSI	Ref.			Ref.		
Yes EOSI	1.27	1.09–1.47	0.003	1.27^a^	1.08–1.51	0.005
Analysis for the subgroup in the lowest wealth quintile						
No EOSI	Ref.			Ref.		
Yes EOSI	0.88	0.71–1.06	0.168	0.95^c^	0.79–1.20	0.808
Analysis for the subgroup in the second lowest wealth quintile						
No EOSI	Ref.			Ref.		
Yes EOSI	1.04	0.78–1.39	0.789	0.97^c^	0.73–1.27	0.796
Analysis for the subgroup in the middle wealth quintile						
No EOSI	Ref.			Ref.		
Yes EOSI	1.46	1.08–1.97	0.013	1.42^c^	1.03–1.97	0.034
Analysis for the subgroup in the second highest wealth quintile						
No EOSI	Ref.			Ref.		
Yes EOSI	1.55	1.02–2.35	0.041	1.49^c^	0.93–2.38	0.095
Analysis for the subgroup in the highest wealth quintile						
No EOSI	Ref.			Ref.		
Yes EOSI	2.27	1.43–3.60	0.001	2.12^c^	1.33–3.39	0.002

a Adjusted for age, marital status, level of education, wealth index, region of origin, area of residence, and contraceptive method.

b Adjusted for age, marital status, level of education, wealth index, region of origin, and contraceptive method.

c Adjusted for age, marital status, level of education, region of origin, and contraceptive method.

EOSI: Early Onset of Sexual Intercourse; STIs: Sexually Transmitted Infections; CPR: Crude Prevalence Ratio; APR: Adjusted Prevalence Ratio; MSP: Multiple Sexual Partners; PR: Prevalence Ratio; CI 95%: 95% Confidence Interval.

## 4. Discussion

### 4.1. Main findings

Using the data from more than 31,000 self-reports by Peruvian women of childbearing age who had already initiated their sexual activity, an association was found between the EOSI and the diagnosis or clinical symptoms of STIs in the last 12 months. Moreover, considering the area of residence, multiple sexual partners, and wealth index, an association between the EOSI and STIs was found only in women living in urban areas, those without a history of multiple sexual partners, those in the middle and highest wealth quintiles, and was marginally statistically significant in the subgroup within the second highest wealth quintile.

### 4.2. Comparison with other studies

Several studies have evaluated the association between the EOSI and STIs, early pregnancies, abortions, and long-term sexual consequences (40). However, to the best of our knowledge, no such studies have been conducted in Latin America, a region where the control of STIs is a persisting challenge (41). In this study, we determined that the EOSI increases the prevalence of STIs by 27% in women of childbearing age compared with those who report initiating sexual activities after the age of 15 years. Similarly, a longitudinal study in the United States of America (USA) (30) found that the EOSI was associated with lifelong STIs and during the last year in young women aged 24–34 years. Miller et al. (42) evaluated sexually active women between the ages of 15 and 44 years in the United States, and found that the prevalence of STIs (gonorrhea and chlamydia) was inversely proportional to the age of sexual activity initiation (13% STI prevalence at <15 years, 7% at 15–16 years, 5.3% at 17–18 years, and 3.4% at >19 years). In contrast, a study conducted in Spain (43) found no association between the EOSI and STIs in women between the ages of 18 and 70 years. However, the definition of the EOSI in the study was beginning sexual intercourse before the age of 18 years, whereas in this study, the EOSI is defined as the initiation of sexual activities before the age of 15 years.

An association between the EOSI and STIs were found in women with a history of having <2 sexual partners during the last 12 months. Similarly, in a sample of adults aged 15–49 years whose average number of sexual partners was <2 during their lifetime, the EOSI was observed to increase the STI odds in the last year (44) (OR: 1.41, 95% CI: 1.26–1.58). Additionally, the individuals with an EOSI who were sexually active with their partner during the last year were less likely to use a condom with their partner than those with a late onset of sexual intercourse. When evaluating the association between the EOSI and STIs according to the area of residence, this study identified the association only in the women living in urban areas. Similarly, Pinzon et al. (45) identified higher STI odds (specifically for syphilis) in Colombian adults living in an urban area, particularly in those who have been sexually active for a longer period than in those who had been sexually active for <20. Although a long period of sexual activity might suggest an EOSI, this is not accurate. The abovementioned study also included an urban population recently displaced from rural areas. In this study, a higher frequency of STIs was observed in the women living in urban areas than in rural areas. Similarly, there have been reports of a higher frequency of EOSI in sexually active Vietnamese women living in urban areas (15). Olakunle and Banougnin found a greater use of modern contraception that did not protect against STIs in Nigerian women aged between 14 and 24 years living in urban areas than in rural areas (46).

In our study, we found that EOSI was associated with STIs in women from the middle wealth quintile upwards. Socioeconomic status has been differently associated with both variables in the literature. A study conducted by Newbern et al. (47) reported that socioeconomic status was a weak to moderate indicator for STIs in American adolescents, while research in Uganda found that living in areas of higher socioeconomic status was associated with a higher risk of STIs (48). Regarding EOSI, the association reported in the literature is inverse, as evidenced in studies conducted in Africa, where young women belonging to higher wealth indexes had a lower risk of EOSI (49, 50).

### 4.3. Interpretation of the results

The EOSI has been associated with having multiple sexual partners, paying for sex, inconsistent use of contraception (44), group and/or casual sex (12), and depression (30). These also represent risk factors of STI transmission, thus supporting its association with the EOSI. An EOSI is hypothesized to cause the proportional increase of HIV cases in adolescent women aged 12–17 years in Peru during the last 5 years (51). Likewise, the increase of the EOSI in Peru (the prevalence of the EOSI was 5.1% in 2000 vs. 6.2% in 2009) (52) and its association with STIs in urban areas could explain the increase in STI cases in women and men from these areas (53). The migration of people from the rural to urban areas also poses a risk of increasing STI cases, with a higher STI risk reported in women migrating from rural to urban areas (54, 55).

Our study also demonstrated an association between the EOSI and STIs in women with <2 sexual partners during the last year, which might be accounted for by the inconsistent use of condoms, leading to a higher risk of STI transmission (55). Furthermore, it is important to highlight that several STIs are chronic and could be previously contracted by the women with an EOSI through transmission by the multiple sexual partners they might have had throughout their lives or prior to the year of evaluation (56), or due to their history of other sexually risky behaviors, such as casual or group sex (12). In addition, a cause of the EOSI in women is sexual abuse at an early age (57), wherein the sexual activities usually occurred without the use of condoms (58). Also, women with a history of sexual abuse at an early age show higher rates of sexual dysfunction (59), which could explain the lower number of sexual partners and chronic STIs.

The association between EOSI and STI was primarily evident in women belonging to a middle-to-high socioeconomic status, which may have several explanations. First, sexual patterns and practices may differ across various socioeconomic levels. For instance, in lower socioeconomic statuses, there is a higher prevalence of sexual relationships within stable and monogamous relationships (60), which could reduce the risk of STI. Second, literature has reported that women with a higher socioeconomic status exhibit increased behavior in seeking health care related to STIs (61). In this regard, limited access to medical care in lower socioeconomic levels could lead to reduced detection and reporting of STI.

### 4.4. Relevance in terms of public health

In 2020, the WHO estimated an incidence of 374 million STIs of bacterial origin (62). Concerning viral STIs, almost 20 million women and girls worldwide were living with HIV in 2020 (63), and almost 500 million people had a herpes infection in 2016 (62). It was estimated that 300 million women were infected with HPV, one of the causes of cervix cancer, the second most frequent type of cancer and the second most lethal among women of childbearing age worldwide (64). On the other hand, the age of beginning sexual activity lowers each year. In addition, the cost of STI treatments throughout people’s lives, according to the beginning of their sexual activity, is approximately calculated to be 16 billion dollars (65). In Peru, one out of five women reports having an EOSI. This study demonstrated an association between the EOSI and STIs in women of childbearing age compared with those who had a late onset of sexual intercourse. Hence, it is imperative to implement preventive measures for STIs in women who are yet to be sexually active and guide their decision-making process regarding this matter by providing them with the necessary tools (66). A few clinical trials of African students report on the relevance of the interventional roles played by schools, parents, communities, and the health system as a group in order to delay the age of sexual activity onset and increase women’s practice of using condoms (67). It is also essential to promote preventive behaviors in adolescents, such as regular attendance to health screenings (53), and to decrease risk factors such as alcohol use (68), smoking, peer pressure, and poor parental supervision (69). Moreover, interventional behavioral counseling (70) (in person or virtually) of sexually active women is recommended to promote safer sex practices; provide training in condom use, problem solving, communication about safer sex, and to assess the person’s risk of acquiring STIs.

### 4.5. Limitations

The present study is cross-sectional, which means establishing causal links between the variables of interest was not possible. Additionally, the STI variable focused on the last 12 months, not accounting for potential previous infections. Other factors associated with STIs, such as the characteristics of sexual partners (71), history of STIs (72), use of antiviral therapy (73), and mental health (74) were not included in this study due to the use of a secondary database. However, our study has strengths, including a large, nationally representative sample, and an analysis that considered area of residence, wealth index, and history of multiple sexual partners. We also adjusted for available confounding variables. Lastly, we did not conduct a mediation analysis because of the lack of certain variables in the database. For example, literature suggests that the use of illicit drugs prior to sexual activity could act as a mediator in the relationship between EOSI and STI (12). Therefore, we believe this should be analyzed in future studies.

## 5. Conclusion

Among Peruvian women aged 15 to 49 years who had initiated their sexual activity, 20% reported an EOSI. In this group, there was a notable association between EOSI and STIs. This association was particularly evident in urban women without a history of multiple sexual partners and in those belonging to a medium to high wealth index. It is recommended to implement measures to prevent EOSI and to provide targeted sexual health counseling for women reporting an EOSI.

## Data availability statement

Publicly available datasets were analyzed in this study. This data can be found at: https://proyectos.inei.gob.pe/microdatos/.

## Author contributions

JP-F and DA-V: conceptualization, investigation, and writing – original draft. MH: investigation and writing – original draft. SC-B: investigation, visualization, and writing – original draft. AL-V: investigation, visualization, writing – original draft, and writing – review and editing. CD-F: methodology, data curation, formal analysis, and writing – original draft. CT-H: conceptualization, methodology, formal analysis, writing – original draft, and supervision. All authors contributed to the article and approved the submitted version.

## Conflict of interest

The authors declare that the research was conducted in the absence of any commercial or financial relationships that could be construed as a potential conflict of interest.

## Publisher’s note

All claims expressed in this article are solely those of the authors and do not necessarily represent those of their affiliated organizations, or those of the publisher, the editors and the reviewers. Any product that may be evaluated in this article, or claim that may be made by its manufacturer, is not guaranteed or endorsed by the publisher.

## Footnotes

^1^ https://proyectos.inei.gob.pe/microdatos/
